# Prosthetic valve endocarditis caused by Staphylococcus capitis: report of 4 cases

**DOI:** 10.1186/1749-8090-6-131

**Published:** 2011-10-07

**Authors:** Tamaki Takano, Yoshinori Ohtsu, Takamitsu Terasaki, Yuko Wada, Jun Amano

**Affiliations:** 1 Shinshu University School of Medicine, Department of Cardiovascular Surgery, 3-1-1 Asahi, Matsumoto 390-8621, Japan

**Keywords:** Prosthetic valve endocarditis, Staphylococcus Capitis, Early surgery, Antibiotics

## Abstract

Although Staphylococcus capitis is considered to be a rare causative organism for prosthetic valve endocarditis, we report 4 such cases that were encountered at our hospital over the past 2 years. Case 1 was a 79-year-old woman who underwent aortic valve replacement with a bioprosthetic valve and presented with fever 24 days later. Transesophageal echocardiography revealed an annular abscess in the aorto-mitral continuity and mild perivalvular regurgitation. We performed emergency surgery 5 days after the diagnosis of prosthetic valve endocarditis was made. Case 2 was a 79-year-old woman presenting with fever 40 days after aortic valve replacement with a bioprosthesis. Transesophageal echocardiography showed vegetation on the valve, and she underwent urgent surgery 2 days after prosthetic valve endocarditis was diagnosed. In case 3, a 76-year-old man presented with fever 53 days after aortic valve replacement with a bioprosthesis. Vegetation on the prosthetic leaflet could be seen by transesophageal echocardiography. He underwent emergency surgery 2 days after the diagnosis of prosthetic valve endocarditis was made. Case 4 was a 68-year-old woman who collapsed at her home 106 days after aortic and mitral valve replacement with bioprosthetic valves. Percutaneous cardiopulmonary support was started immediately after massive mitral regurgitation due to prosthetic valve detachment was revealed by transesophageal echocardiography. She was transferred to our hospital by helicopter and received surgery immediately on arrival. In all cases, we re-implanted another bioprosthesis after removal of the infected valve and annular debridement. All patients recovered without severe complications after 2 months of antibiotic treatment, and none experienced re-infection during 163 to 630 days of observation. Since the time interval between diagnosis of prosthetic valve endocarditis and valve re-replacement ranged from 0 to 5 days, early surgical removal of the infected prosthesis and an appropriate course of antibiotics were attributed to good clinical outcomes in our cases.

## Background

Staphylococcus capitis (S. capitis) is considered to be a rare causative organism of prosthetic valve endocarditis (PVE) since only 4 cases of PVE caused by S. capitis have been reported to date [1-3]. This bacterium is a subtype of coagulase-negative staphylococci (CoNS) and thus produces biofilm, which confers tolerance to disinfectants during surgery. Unlike most CoNS, however, the adhesion ability of S. capitis to foreign body surfaces is low [4,5]. Nonetheless, we report here 4 cases of PVE caused by S. capitis that were encountered at our hospital over the past 2 years.

## Case 1

**Figure 1:**
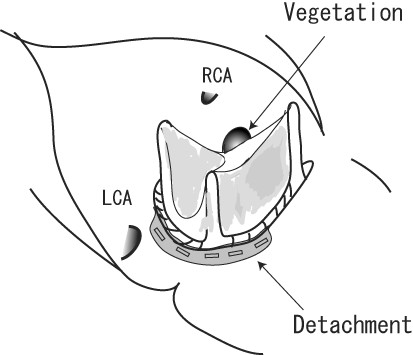
**Operative Findings in Case 1**. Yellowish-white film covered the entire Carpentier-Edwards Magna bioprosthesis, and vegetation located on the right coronary cusp. Valve dehiscence was found at the commissure between the left and non-coronary cusp.

**Figure 2:**
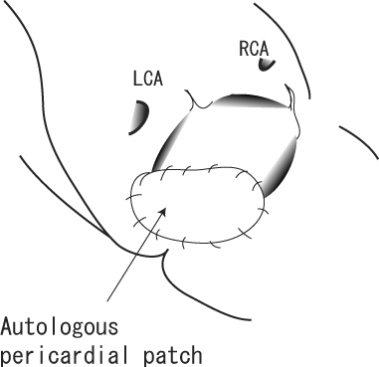
**Operative Findings in Case 1**. Intimal defect was repaired with autologous pericardial patch after the debridement.

A 79-year-old woman underwent aortic valve replacement with a Carpentier-Edwards Magna bioprosthetic valve (Edwards Lifesciences, Irvine, CA) for aortic stenosis. She presented with a fever of over 38°C 24 days after the procedure (Table 1). The first blood culture showed no evidence of bacterial growth, but S. capitis was detected in the second examination. Intravenous administration of gentamicin (GM) was started, which was later changed to abekamicin due to its susceptibility (Table 2). Transesophageal echocardiography (TEE) revealed an annular abscess in the aorto-mitral continuity and mild perivalvular regurgitation. We performed emergency surgery 5 days after the diagnosis of PVE was made. The aortic bioprosthesis was fully covered with a yellowish-white film, and vegetation was seen on the right coronary cusp. Valve dehiscence had occurred around the commissure between the left and non-coronary cusps (Figure 1). The prosthetic valve was removed and the aortic annulus debrided. The intimal defect around the commissure was repaired after debridement with an autologous pericardial patch (Figure 2). A Medtronic Mosaic porcine valve (21 mm) (Medtronic, Minneapolis, MN) was implanted in a supra-annular fashion with horizontal mattress sutures from the left ventricle to the ascending aorta, except for 5 sutures that were passed through the aortic wall and pericardial patch at the intimal defect. Cardiopulmonary bypass was weaned off without difficulty and post-operative course was uneventful. Intravenous vancomycin (VCM) and oral minomycin (MINO) were administered for 2 months after the aortic valve re-replacement (Table 3). We directly contacted with the patients by phone, and no signs of infection were seen during 630 days of follow-up.

**Table 1:** Patient characteristics

	Age (y.o.)	Sex	PVE onset from the first operation (Days)	First valve operation	Surgical Indication	Fever at admission	Heart failure	Embolic Event	Re-operation from the PVE diagnosis (days)	Re-operation from the fever onset (days)
**Case 1**	79	F	24	AVR (Biological)	Annular abscess Regurgitation	+	-	-	5	12

**Case 2**	79	F	40	AVR (Biological)	Vegetation	+	-	-	2	14

**Case 3**	76	M	53	AVR (Biological)	Vegetation	+	-	-	2	8

**Case 4**	68	F	106	MVR (Biological) AVR (Biological)	Regurgitation Shock	+	+	-	0	8

**Mean**	76 ± 5.2		56 ± 36						2.3 ± 2.1	10.5 ± 3

PVE; prosthetic valve endocarditis, F; female, M; male AVR; aortic valve replacement, MVR; mitral valve replacement

**Table 2:** Antibiotics Susceptibility

	PCG	MPIPC	CEZ	IPM	GM	ABK	EM	CLDM	MINO	LVFX	FOM	VCM	ST	TEIC	LZD
**Case 1**	> = 0.5	> = 4	< = 4	< = 1	8	< = 1	< = 0.25	< = 0.25	< = 0. 5	0.5	> = 128	< = 1	< = 10	< = 0.5	N/A

**Case 2**	> 8	> 2	> 16	2	> 8	< = 1	0.5	< = 0.5	< = 1	1	> 16	< = 2	< = 2	< = 2	< = 2

**Case 3**	> = 0.5	> = 4	8	< 1	8	< = 1	< = 0.25	< = 0.25	< = 0. 5	1	> = 128	1	N/A	< = 0.5	2

**Case 4**	> 8	> 2	> 16	4	> 8	< = 1	< = 0.25	< = 0.5	< = 1	< = 0.5	> 16	< = 2	< = 2	< = 2	< = 2

PCG; penicillin G, MPIPC; oxacillin, CEZ; cephazolin, IPM; imipenem, GM; gentamicin, ABK; arbekacin, EM; erythromycin, CLDM; clindamycin, MINO; minocycline, LVFX; levofloxacin, FOM; fosfomycin, VCM; vancomycin, ST; sulfamethoxazole-trimethoprim, TEIC; teicoplanin, LZD; linezolid, N/A; not available

**Table 3:** Re-operation procedure, Antibiotics and Re-infection

	Annular abscess Valve dehiscence	Re-operation Procedure	Prosthesis	Intravenous antibiotics	Oral antibiotics	Observation period (days)	Survive Re-infection
Case 1	+ -	Abscess isolation AVR	Biological →Biological	AMK→ LZD→TEIC	MINO	630	+ -

Case 2	- -	AVR	Biological →Biological	VCM	MINO	332	+ -

Case 3	+ +	Valve annuls reconstruction AVR	Biological →Biological	TEIC→VCM→LZD	LVFX	224	+ -

Case 4	+ +	Valve annuls reconstruction MVR	Biological →Biological	VCM+GM	LVFX	163	+ -

AVR; aortic valve replacement, AMK; Amoxicillin, LZD; linezolid, TEIC; teicoplanin, MINO; minocycline, VCM; vancomycin, LVFX; levofloxacin, MVR; mitral valve replacement, GM; gentamicin.

## Case 2

**Figure 3:**
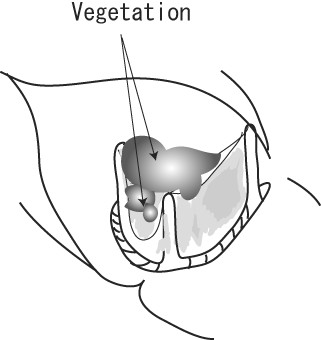
**Operative Findings in Case 2**. Carpentier-Edwards Magna valve was covered by white and yellowish thin film, and vegetations were attached on the stent and prosthetic leaflet.

A 79-year-old woman suffering from aortic stenosis underwent aortic valve replacement with a Carpentier-Edwards Magna bioprosthetic valve. She had a fever of over 38°C and complained of chills 40 days after the procedure (Table 1). All three blood cultures that were taken revealed S. capitis, and so intravenous administration of VCM and rifampicin was commenced (Table 2). TEE revealed vegetation on the bioprosthesis, which gradually increased in size. Aortic valve re-replacement was performed 2 days after the diagnosis of PVE was made and 14 days after fever onset. A yellowish-white film covered the whole bioprosthetic valve, and vegetation was found on the stent and prosthetic leaflet at a maximum size of 20 mm in diameter (Figure 3). Neither valve dehiscence nor annular abscess was observed. A Medtronic Mosaic porcine valve (19 mm) was inserted after the Magna valve and biofilm were removed. She presented with transient dysarthria after the surgery, but recovered fully within a month. Intravenous VCM was continued for 2 months after the re-replacement, and MINO was given orally after hospital discharge (Table 3). There were no signs of infection were observed during 332 days of follow-up.

## Case 3

**Figure 4:**
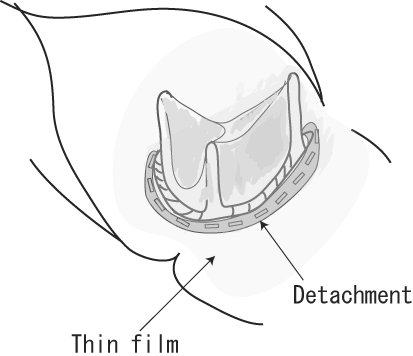
**Operative Findings in Case 3**. The bioprosthetic valve was totally detached from the annulus.

**Figure 5:**
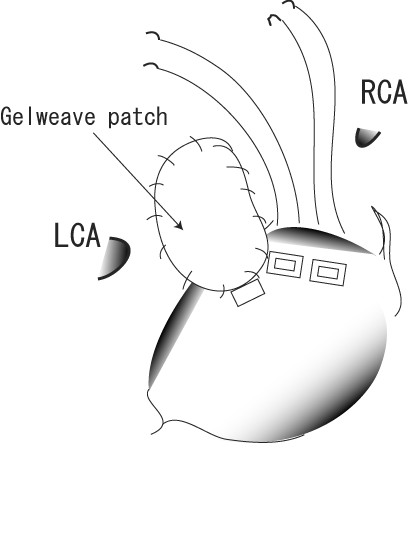
**Operative Findings in Case 3**. The defect of the aortic annulus and aortic wall was repaired with Gelweave graft patch after annular abscess debridement.

A 76-year-old man was hospitalized for a fever of over 38°C and general malaise 53 days after aortic valve replacement with a Carpentier-Edwards Magna bioprosthesis (Table 1). TEE revealed a thickened bioprosthetic leaflet covered with vegetation. S. capitis was identified by a blood culture, and a pseudoaneurysm at the edge of the aortotomy closure was seen in a chest CT scan. We performed emergency surgery 2 days after the diagnosis of PVE was made and 8 days after fever onset. As the Magna bioprosthesis had detached from the annulus (Figure 4), the valve was removed and the annular abscess debrided. A Medtronic Mosaic porcine valve (21 mm) was implanted after an aortic wall defect in the aortic annulus caused by the debridement was repaired with a Gelweave graft patch (Terumo Corporation, Tokyo, Japan) (Figure 5). The pseudoaneurysm of the ascending aorta was resected and re-constructed with a Gelweave graft. He was sequentially administered intravenous teicoplanin, VCM, and linezolid (LZD) due to liver dysfunction (Table 3), and was discharged from the hospital 2 months after the re-replacement without any complications. He was followed up at the outpatient clinic, and no signs of infection were seen during 224 days after the surgery.

## Case 4

**Figure 6:**
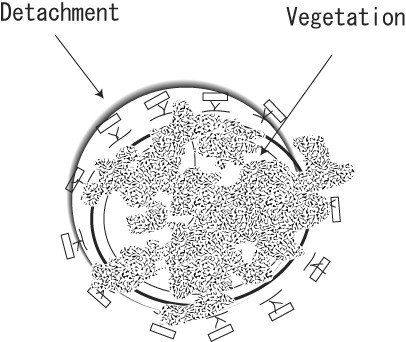
**Operative Findings in Case 4**. Yellowish white film covered the whole mitral valve, and valve detachment had occurred at the 1/3 of the annulus.

A 68-year-old woman collapsed in her home 106 days after aortic and mitral valve replacement with a Carpentier-Edwards bioprosthesis (Edwards Lifesciences, Irvine, CA) (Table 1). She was found in a shock-like state with a systolic blood pressure of 60 mmHg, immeasurable blood oxygen saturation, and decreased consciousness. As TTE showed massive mitral regurgitation due to prosthetic valve detachment, percutaneous cardiopulmonary support was started immediately. She was airlifted to our hospital, and an emergency surgery was performed. A yellowish-white film covered the whole mitral valve, which had become detached at 1/3 of the annulus. An abscess was found in the remaining annulus (Figure 6). Mitral valve re-implantation was performed with a Medtronic Mosaic bioprosthesis (25 mm) after entire annular debridement and partial reconstruction of the annular defect with bovine pericardium. Intravenous VCM and GM were administered for 2 months after the valve re-implantation, and the patient underwent cholecystectomy for cholecystolithiasis that had been diagnosed before the initial valve replacement (Table 3). She was discharged from the hospital without any neural deficits and is leading a normal daily life. We directly contacted with the patients by phone, and no signs of infection were seen during 163 days of follow-up.

## Discussion

Although several reports of native valve endocarditis caused by S. capitis [6,7] exist, there have been only 4 published cases of S. capitis causing PVE since 1996 [2]. This paper describes 4 additional cases of PVE caused by S. capitis encountered at our hospital that revealed several important clinical findings.

S. capitis is a subtype of CoNS that is characteristically novobiocin-sensitive, aerobic, and hemolysis-positive. However, this bacterium lacks alkaline phosphatase activity, which differentiates it from S. epidermidis [8]. S. capitis did not account for any incidences of bacteremia, intravenous catheter-associated infection, prosthetic valve infection, cerebrospinal fluid infection, or peritonitis, although it was detected in blood or intravenous catheters in 4% of patients without these conditions [4]. The most common causative organism of PVE is Staphylococcus aureus, followed by the CoNS Enterococcus and Streptococcus viridans, according to a recent report [9].

The ability of surface-adherent growth on prosthetic devices is considered to be potentially important in causing disease [4,10]. S. capitis is known to have weak adhesion to smooth surfaces, unlike most other CoNS, such as S. epidermidis [4,5]. The virulence of CoNS is mainly attributed to their adhesion ability to smooth surfaces, biofilm production, and secretion of exoenzymes. An annular abscess was found in 3 of 4 cases and prosthetic valve dehiscence occurred 2 of 4 cases in the present report (Table 3). These findings demonstrate that S. capitis may still cause fatal destruction of the prosthetic valve annulus despite its relatively weak adhesion ability to foreign body surfaces.

Treatment of PVE remains a challenge. The in-hospital mortality rate of PVE is 21-28.4% [9,11,12], even with correct evaluation of the prosthetic valve by TEE in suspected patients. It is believed that preoperative status and complications are strongly related to the early mortality in PVE [11]; preoperative catecholamine, dialysis, pulmonary edema, ventilation, and renal insufficiency are all predictors for 30-day mortality. In the present series, all patients survived and none experienced re-infection during 163 to 630 days of observation (Table 3). Urgent surgery is recommended for patients with complicating PVE [12]. We performed re-operations from 0 to 5 days after PVE diagnosis for a mean time interval between fever onset and surgery of 10.5 ± 3 days, which was considerably shorter than the 15 days reported elsewhere [12]. This early surgical intervention may be considered to be attributed to good clinical outcome.

It is important to distinguish causative organism from skin flora because S. capitis may colonize on skin like other CoNS. We repeated blood culture at least three times and detected only S. capitis in the each case. We therefore considered S. capitis as the causative organism for PVE. Infection route of S. capitis could not be clearly known because 1 of 4 cases were late PVE and any predisposing factor was not observed in the previous reports [1-3] although 3 of 4 presenting cases were early PVE, which might speculate contamination during the initial valve replacement.

S. capitis was successfully treated in all cases with similar susceptibility and sensitivity to VCM, TEIC, and LZD (Table 2), although decreased susceptibility of CoNS to VCM and TEIC has been reported [13]. Thus, the antibiotic treatment course used in our patients may be useful for future cases of PVE caused by S. capitis, as well as for other culture-negative bacterial PVE, which accounts for 11.2% of all cases [9], whereas CEZ was used as perioperative prophylaxis in the initial valve replacement of all the presenting cases. Our findings also indicate a need to reassess the virulent nature of S. capitis, especially with regard to bioprostheses.

## Conclusion

We experienced 4 cases of PVE caused by S. capitis. Early surgical removal of the infected prosthesis and administration of appropriate antibiotics may play important roles in successful PVE treatment.

## Consent

Written informed consent was obtained from the patients for publication of this Case report. Copies of the written consent forms are available for review by the Editor-in-Chief of this journal.

## List of abbreviations

CoNS: coagulase-negative staphylococci; GM: gentamicin; LZD: linezolid; MINO: minomycin; PVE: prosthetic valve endocarditis; S. capitis: Staphylococcus capitis; TEE: Transesophageal echocardiography; VCM: vancomycin.

## Competing interests

All the authors have read the manuscript and have approved of its submission. The authors report no conflicts of interest.

## Authors' contributions

TT presented design of the case report and completed the manuscript. YO, TT and YW are in charge of patient care. JA directed all the work. All authors read and approved the final manuscript.
